# Absorbable Suture as an Apical Matrix in Single Visit Apexification with Mineral Trioxide Aggregate

**DOI:** 10.1155/2016/4505093

**Published:** 2016-12-12

**Authors:** Ayush Goyal, Vineeta Nikhil, Padmanabh Jha

**Affiliations:** Department of Conservative Dentistry & Endodontics, Subharti Dental College, Meerut, Uttar Pradesh, India

## Abstract

Several procedures have been recommended to induce the root end barrier formation in teeth with open apices. Conventional treatment for such cases will require many appointments with an average duration of 12.9 months. During this period, the root canal is susceptible to reinfection from around the provisional restoration, which may promote apical periodontitis and arrest of apical repair. Mineral trioxide aggregate (MTA) has been successfully used for one visit apexification wherein the root canal can be obturated within 24 hours after placement of MTA. Using a matrix prior to the placement of MTA avoids its extrusion, reduces leakage in the sealing material, and allows favorable response of the periapical tissues. This report presents a case of apexification where an absorbable suture was used as an apical matrix. Use of an absorbable suture circumvents all the problems associated with other conventional materials.* Conclusion*. Placement of the matrix made from the suture material is predictable and is easily positioned at the apex and the length can be adjusted as required. 10-month follow-up of the case shows resorbed matrix and bone healing in the periapical region. The patient was asymptomatic during the whole follow-up period and tooth exhibited mobility within physiologic limits and was functioning normally.

## 1. Introduction

When teeth with incomplete root formation suffer trauma, the root development ceases and the apical closure cannot be achieved. Luxation injuries appear to be associated with greatest risk of incomplete root development and according to J. O. Andreasen and F. M. Andreasen, about 15 to 59% teeth lose their vitality [1]. The maxillary central incisor is the most common tooth affected in both dentitions [1].

Root canal treatment of a tooth with immature apex is an arduous task because of large size of the canal, thin and fragile roots, and absence of an apical barrier against which to condense the obturating material. Apexification is a treatment modality which induces a calcified barrier in teeth with immature apices. The purpose of this barrier is twofold [2], (a) to prevent the passage of toxins and bacteria from the root canal into the periapical tissues and (b) to allow the compaction of the root filling material.

Calcium hydroxide has been a widely reported and probably the most studied material for apexification. Granath was the first to report the use of the material for apical closure [3]. Though its efficiency has been reported by many authors, it has certain disadvantages, like unpredictability in the duration of treatment (average duration is 12.9 months) [4], patient compliance due to multiple recalls, and increased risk of root fracture following several calcium hydroxide dressings.

It is possible to circumvent all the above problems with MTA which has achieved widespread acceptance in this field. The main advantages of MTA are (a) reduction in the treatment time leading to better patient compliance, (b) decreased risk of fracture of root as the tooth can be permanently restored with minimal delay, and (c) minimal alteration of mechanical properties of dentin.

Although MTA is reported to be biocompatible, overfilling of root canals with MTA yielded significantly inferior results compared to obturation performed at the limit of the cemental canal [5]. Using a matrix prior to the placement of MTA avoids its extrusion, reduces leakage in the sealing material, and allows favorable response of the periapical tissues [6]. Several materials have been recommended to create a matrix, like hydroxyapatite-based materials, resorbable collagen, platelet-rich fibrin, and calcium sulphate [7–9]. All these materials share a common disadvantage that once placed, their position cannot be adjusted.

The present case demonstrates the use of an absorbable suture that was modified to form an apical matrix in a single visit apexification procedure with MTA with a 10-month follow-up.

## 2. Case Report

**Figure 1:**
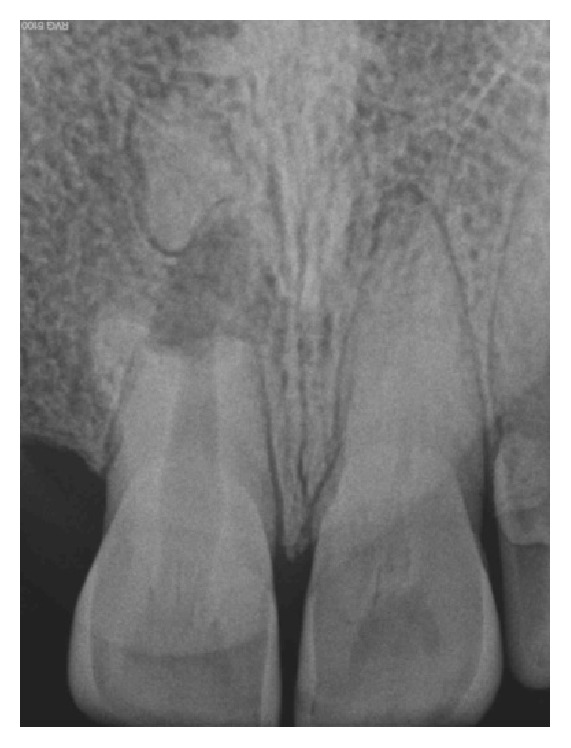
Preoperative intraoral periapical (IOPA) radiograph showing open apex in relation to 11.

A 19-year-old male patient reported to the Department of Conservative Dentistry and Endodontics, Subharti Dental College, Meerut, with a chief complaint of pain in relation to his upper front teeth region since few weeks. On examination, it was found that the tooth in question was maxillary right central incisor (11). Pain was moderate in intensity, nonradiating, and aggravated on biting. Clinical examination revealed an Ellis class I fracture in relation to 11. The patient reported a history of trauma around 5 years back. Tooth was moderately tender on percussion. Radiograph in relation to 11 showed a wide-open apex along with an area of periapical rarefaction (Figure 1). Pulp sensibility tests in relation to 11 did not elicit any response. Cold test was performed using Endo Ice® (Coltène/Whaledent Inc., Cuyahoga Falls, USA). The tooth did not respond to electric pulp testing also. Based on clinical and radiographic findings, a diagnosis of pulpal necrosis with symptomatic apical periodontitis was made.

The crown-root ratio of the subject tooth was 1 : 1 (approx.). Though the crown-root ratio may be considered inadequate by some clinicians, the tooth exhibited no abnormal mobility and the patient and his parents were not willing for extraction (at any cost!). It was decided to carry out single visit apexification using MTA.

**Figure 2:**
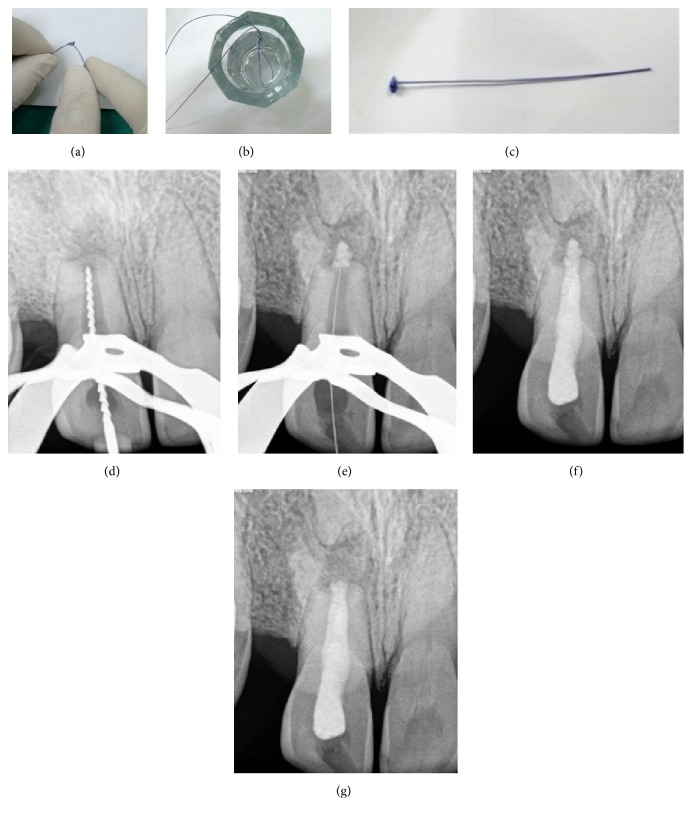
(a)–(c) Fabrication of the matrix. (a) Formation of the “apical matrix” by tying a knot with the suture material. (b) The suture material was kept in the radio-opaque dye for 15 minutes. (c) “Modified suture apical matrix” ready for placement in the root canal. (d)–(g) Intracanal procedures. (d) Working length radiograph. (e) Apical matrix was placed at the apex and the free end was left outside for adjustment. (f) Radiograph taken after placement of MTA, obturation, and coronal restoration with composite. (g) 3-month recall shows absorption of the “apical matrix.”

After administration of local anaesthesia, Lignocaine HCl with adrenaline 1 : 80000 (Lignox 2% A, Indoco Remedies Ltd., Mumbai, India) and rubber dam isolation, access was opened. The canal was debrided using Hedström files followed by copious irrigation with 3% sodium hypochlorite (Novodent Equipments & Materials Ltd., Mumbai, India). Working length was determined radiographically with a file placed in the canal (Figure 2(d)). The canal was dried using sterile paper points (Meta® BioMed, Korea). Braided coated polyglactin-based, 3-0 absorbable suture material VICRYL™ (Johnson and Johnson Ltd., Aurangabad, India) was used for the formation of apical matrix. The suture was tied to form a knot and was tied 2 more times to form a thicker knot (Figure 2(a)). The thickness of the knot can be approximately determined by gauging the apex with a large size file. This knot would serve as an apical matrix and the free end of the suture material can be used to adjust its length. The suture material was placed in an iopamidol solution (61%) (ISOVUE®-300, Bracco Diagnostics, Italy) for 15 minutes to make it radiopaque (Figures 2(b) and 2(c)). The “matrix” was then placed in the canal and was pushed to position it at the apex using a set of preselected hand pluggers. The position of the matrix was confirmed radiographically (Figure 2(e)). Once the matrix was in position, MTA (White Pro-Root MTA®, Dentsply Maillefer, Ballaigues, Switzerland) was mixed according to the manufacturer's instructions to a thick creamy consistency and placed in the canal using an MTA carrier (Messing Gun®, Produits Dentaires, Vevey, Switzerland). MTA was condensed with the butt end of sterile damp paper points to form a 3 mm MTA plug. After this, free end of the suture was cut with a thin scissor as close as possible to the MTA plug and another 1 mm MTA plug was packed. This was done to avoid any suture material remaining inside the canal which may result in a longitudinal filling defect at the tooth-restoration interface. A moist cotton pellet was placed in the canal and the access cavity was temporized with Cavit™ G (3M ESPE, Neuss, Germany). The patient was recalled next day and the root canal was obturated using lateral condensation technique with gutta percha (Meta® BioMed, Korea) and AH Plus sealer (Dentsply Detrey GmbH, Germany). Finally, the access cavity was restored with resin composite (Figure 2(f)). The patient was recalled 2 weeks later and demonstrated no clinical signs and symptoms. At 3-month recall, the tooth exhibited mobility within physiologic limits and no evidence of periodontal pockets and was functioning normally. Radiographic examination revealed ongoing resorption of the apical matrix (Figure 2(g)). 10-month follow-up of the case shows resorbed matrix and bone healing in the periapical region (Figure 5).

## 3. Discussion

Apexification is defined as “a method to induce a calcified barrier in a root with an open apex or the continued apical development of an incomplete root in teeth with necrotic pulp” [10]. Traditionally, calcium hydroxide has been used extensively for apexification. Dominguez Reyes et al. [4] reported 100% success rate with calcium hydroxide. However, there are a number of factors which might lead to failure of the apexification procedure in this technique [2]; for example, high pH (12.7) of calcium hydroxide can induce a necrotic zone in the periapical area, risk of contamination of the root canal space since a permanent restoration cannot be placed until the treatment is complete, and resultant decrease in the strength of roots which ultimately may lead to fracture even before the treatment is complete. On the other hand, in a single visit apexification procedure with MTA, obturation and coronal restoration can be done within 24 hours after the placement of MTA. This is a definite advantage when compared to the traditional technique of apexification.

In a prospective study by Simon et al. [2], forty-three cases were followed up for one year after one-visit apexification procedure with MTA. The authors reported a high success rate (81%) with this technique and concluded that apexification with MTA is a predictable and reproducible procedure. As mentioned above, use of an internal matrix makes the compaction of MTA easier and placement predictable.

Various materials, like platelet-rich fibrin, calcium sulphate, hydroxyapatite, and resorbable collagen, have been used as internal matrix. However, all these materials possess certain disadvantages. Calcium sulphate has a short setting time of 1-2 minutes [7]. This is a major drawback of this material. If not placed correctly inside the canal, there is hardly any time to readjust it. Secondly, it requires placement using specialized devices like Messing gun or Dovgan carriers. Thirdly, care has to be taken while placement so that calcium sulphate does not contact the root canal walls as it interferes with the close adaptation of MTA. Lastly, the tip of the carrier has to be cleaned immediately after placement, otherwise calcium sulphate sets and is difficult to remove. Placement of collagen membranes is technique sensitive and requires high level of accuracy in positioning [6]. Hydroxyapatite is difficult to manipulate, is granular in consistency, has poor adaptability to the walls, and does not set [11]. Moreover, it is an expensive material. Platelet-rich fibrin, though it has shown promising results in various case reports, has an inherent disadvantage of being radiolucent. Radiographic confirmation can only be done after the apical plug has been placed with a barrier material.

Coated VICRYL (polyglactin 910) suture is a synthetic absorbable sterile surgical suture composed of a copolymer made from 90% glycolide and 10% L-lactide [12]. It is commonly used in subcutaneous, intracutaneous, abdominal, and thoracic surgeries. Coated VICRYL suture is prepared by coating the suture material with a mixture composed of equal parts of copolymer of glycolide and lactide (polyglactin 370) and calcium stearate [13]. Calcium stearate is a salt of calcium and stearic acid, both of which are present in the body and constantly metabolized and excreted [13]. Coated VICRYL has been found to be nonantigenic and nonpyrogenic and elicit only a mild tissue reaction during absorption [14, 15]. Absorption of coated VICRYL suture is complete between 56 and 70 days by hydrolysis [13]. Lactide and glycolide acids are readily eliminated from the body, primarily in urine [13]. Manufacturers of VICRYL suture material state that the absorption of the suture material may be accelerated if the suture is contaminated with blood or water prior to suturing procedure. Since the suture material was placed in a radio-opaque dye for 15 minutes, it is possible that the absorption process would have initiated earlier. However, since the patient was recalled only after 3 months, it is difficult to quote the exact time of absorption.

The certain advantages of this technique over conventional techniques and materials mentioned previously are as follows: (a) easy “fabrication” of the matrix, (b) length being adjusted as required using the free end of the suture, (c) simple placement technique using pluggers, (d) easy availability of absorbable sutures, (e) being inexpensive, and (f) VICRYL being not a newly introduced suture material; it has been in use for surgeries for more than three decades. Since a suture material was modified to form a knot which in turn serves as a matrix, the authors would like to term this technique “modified suture apical matrix” technique.

The dye iopamidol was used to impart radiopacity to the matrix. Iopamidol has been used as diagnostic agent for clinical CT protocols since 1981 [16]. High water solubility coupled with very low toxicity makes it an ideal contrast agent for various diagnostic purposes [16]. It has been used for angiography throughout cardiovascular system, pediatric angiocardiography, selective visceral arteriography and aortography, phlebography, adult and pediatric intravenous excretory urography, and intravenous adult and pediatric Contrast Enhancement of Computed Tomographic (CECT) Head and Body Imaging [17]. It is available in various concentrations, from 200 to 370 mg/mL. ISOVUE®-300 used in the present case contains 300 mg organically bound iodine per mL. Presence of iodine atoms endows iopamidol molecule with high X-ray radiopacity [16].

**Figure 3:**
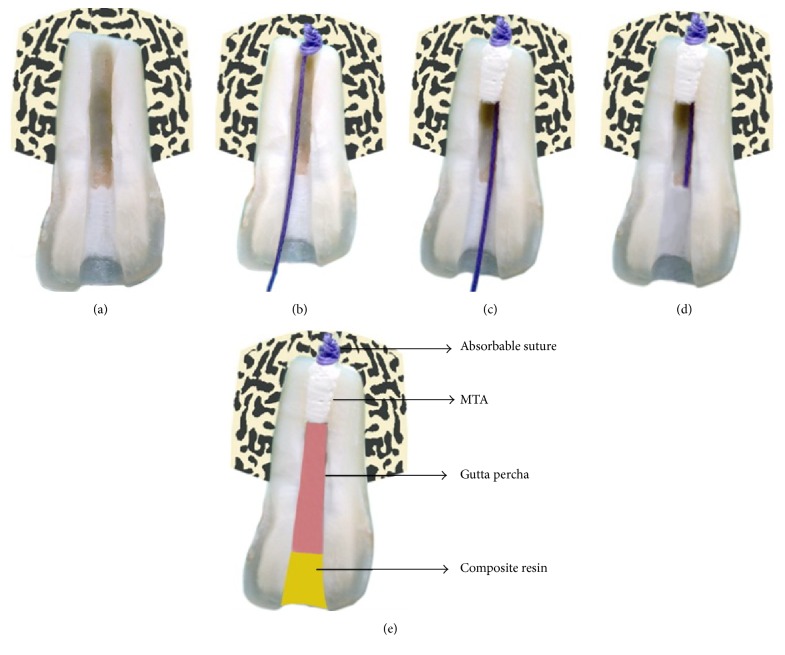
Modified suture apical matrix concept. (a) Tooth with immature apex. (b) Suture matrix placed at the apex. (c) MTA is condensed against the matrix. (d) The free end of the suture is cut. (e) Obturation is done followed by a permanent coronal restoration.

Iodine and iodinated contrast agents have been shown to cross the placenta [18] and have been reported to pass into breast milk [19]. Hence, when used as a contrast media, it is contraindicated for pregnant patients and lactating mothers. However, when used as an apical matrix for endodontic purpose, it is unclear whether it should be used in such patients or not. It is best to avoid iopamidol until further data is available on the same. Also, iopamidol is advised against use in cases of active infection. However, most data available on iopamidol is based on its indications as a contrast media. Further studies could probably throw some light on this subject. Iopamidol is eliminated by the body, primarily in urine [17]. McKinstry et al. [17] have reported a urinary recovery of 90% or more of the dose within 72 to 96 hours after intravenous injection of iopamidol.  Figure 3 shows a schematic representation of the whole procedure.

**Figure 4:**
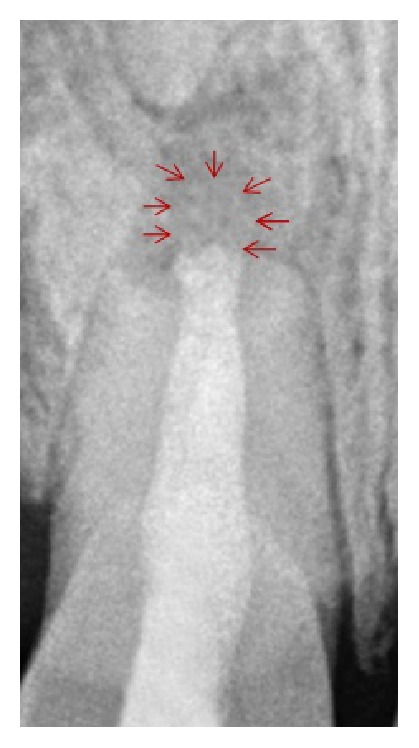
Silhouette of the resorbed suture material when the image is zoomed in to 140%.

**Figure 5:**
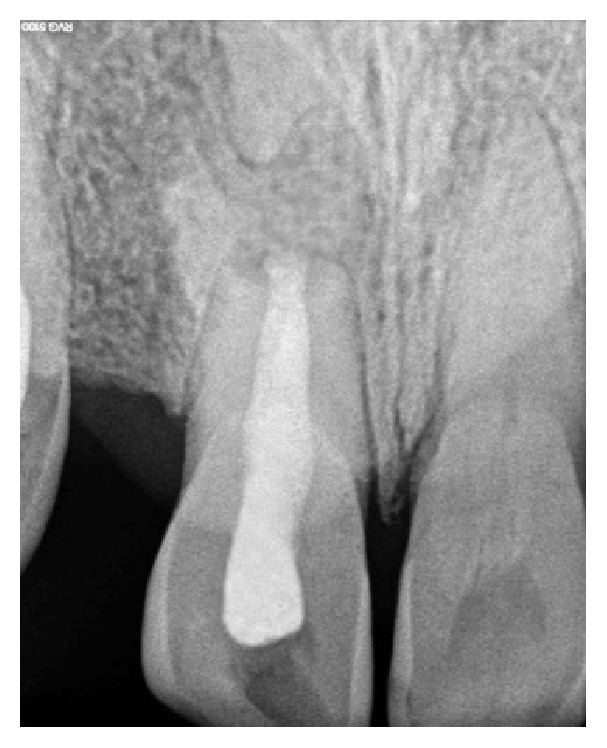
10-month follow-up of the case shows completely resorbed matrix and bone healing in the periapical area.

An unusual finding in the present case was the wide-open apex even though the tooth was traumatized at 14 years of age, that is, after the root formation was complete for central incisors. A careful look at the preoperative radiograph will reveal a “root-tip” shaped radiopacity about 5 mm from the apex of the tooth. It is the opinion of the authors that the trauma must have caused the tooth to suffer a horizontal root fracture in addition to intrusion. With time, the intruded tooth may have erupted until it reached its original position in the arch while leaving the root tip behind. The radiopacity is surrounded by a thin radiolucency very similar to periodontal ligament. This supports the fact that radiopacity may in fact be the root tip. One might feel that the data provided by the patient regarding the time of trauma may be incorrect. But careful history was taken before arriving at any conclusion and the patient and his parents were most certain of the time of trauma. Another finding that the authors would like to share is a silhouette of the resorbed suture material when the image is zoomed in to 140% (Figure 4). It is possible that scattered radio-opaque filaments may have imparted this appearance. It may be speculated that there is some effect on the absorption rate of dye when combined with suture material. It remains unclear whether this finding is of any clinical significance.

The technique mentioned in this report is easy to carry out and very importantly allows for adjustment of the position during the placement. The authors encourage further research on this subject using different suture materials like, for example, Coated VICRYL® Plus Antibacterial (polyglactin 910) Suture which has an antibacterial coating on it. Research investigating the reaction of periapical tissues to the suture material and the radio-opaque dye is further encouraged.

## 4. Conclusion

The technique mentioned in this report is new. However, the materials used for it are not. The suture material and the radio-opaque die have been approved by the Food and Drug Administration (FDA) and have been in use for several years. The technique makes it possible for the clinicians to bypass certain disadvantages encountered with other techniques mentioned in the literature. More case reports and long-term follow-up periods with this technique are required for it to establish a firm foot in the field of endodontics.
